# Transparency in public pharmaceutical sector: the key informants’ perceptions from a developing country

**DOI:** 10.1186/s12913-021-07319-x

**Published:** 2021-12-07

**Authors:** Atefeh Esfandiari, Vahid Yazdi-Feyzabadi, Leila Zarei, Arash Rashidian, Hedayat Salari

**Affiliations:** 1 Department of Health Policy & Management, School of Medicine, Bushehr University of Medical Sciences, Bushehr, Iran; 2 Health Services Management Research Center, Institute for Futures Studies in Health, Kerman University of Medical Sciences, Kerman, Iran; 3 Department of Health Management, Policy and Economics, Faculty of Management and Medical Information Sciences, Kerman University of Medical Sciences, Kerman, Iran; 4 Health Policy Research Center, Institute of Health, Shiraz University of Medical Sciences, Shiraz, Fars Iran; 5 Department of Health Management and Economics, School of Public Health, Tehran University of Medical Sciences, Tehran, Iran; 6 Information, Evidence and Research Department, Eastern Mediterranean Regional Office, World Health Organization, Cairo, Egypt

**Keywords:** Transparency, Pharmaceutical sector, Stewardship, Evidence, Public sector

## Abstract

**Background:**

Policymaking in the pharmaceutical sector plays a pivotal role in achieving the health systems’ goals. Transparency in the pharmaceutical policy could increase confidence in decision-making processes. This study aims to assess transparency in the public pharmaceutical sector of Iran.

**Methods:**

This qualitative study with a content analysis approach was conducted in 2017 using the World Health Organization tool to explore pharmaceutical transparency. The perceptions of the various stakeholders of the health system through semi-structured interviews with a maximum variation of stakeholders were obtained in eight functions, including registration, licensing, inspection, promotion, clinical trials, selection, procurement, and distribution of medicines.

**Results:**

There are some problems in two main categories: (1) General problems, including lack of transparency, conflict of interest, centralization, and monopoly. (2) Ethical problems include illegal payments, gifts, bribes, conflicts of interest, hidden power, hoarding, relationship-oriented behavior, medicine trafficking, and counterfeit medicine. Suggested solutions include evidence-based decision-making, the use of transparent and accountable processes, standardization, needs assessment, declaring a conflict of interest, skilled human resources, and tracking prescription.

**Conclusion:**

Despite the development of effective pharmaceutical policy in the health care system and government interventions for the control of the market, in some functions, reviewing the pharmaceutical policy is essential. Additionally, declaring a conflict of interest statement must be at the core of policy development to provide greater transparency.

**Supplementary Information:**

The online version contains supplementary material available at 10.1186/s12913-021-07319-x.

## Background

Pharmaceutical products are one of the critical technologies, and the pharmaceutical sector is one of the main components of health care systems worldwide [1]. However, the literature raised that the availability and affordability of medicines, even essential medicines, are deficient in low-income and middle-income countries (LMICs) [2–6]. On the other hand, drugs account for 20–60% of health care expenditures in developing and transition countries, which is higher than the Organization for Economic Co-operation and Development (OECD) countries (about 18%) [7]. More than 90% of the population in developing countries buy medicines from out-of-pocket (OoP) payments [4]. In addition, the high value of the pharmaceutical market, the complex processes and various steps involved in the pharmaceutical supply chain, and the complex mix of actors with diverse responsibilities make the pharmaceutical sector is increasingly susceptible to corruption and unethical practices [8].

Corruption is one of several factors that may hinder access to pharmaceuticals [9]. A high level of transparency and accountability is critical for minimizing opportunities for fraud and leakage. So, a growing number of public health officials in ministries of health and national medicines regulatory authorities recognize the need for their institutions and personnel to work in a transparent and accountable environment [10].

In Iran, the Ministry of Health and Medical Education (MOHME) is responsible for policy-making, implementation, and monitoring of the national-level health policies [11, 12]. In the pharmaceutical sector, this responsibility has been delegated to Iran Food and Drug Administration (IFDA) [13, 14]. The registration of pharmaceutical products in the National Drug List (NDL), licensing policy, the proposal to allocate preferential currency, subsidizing certain drugs, emergency import, and pricing policy are just some examples of the IFDA action in Iran [13, 14].

With the reinforcement of internal drug production capabilities, the Iranian pharmaceutical industry tries to get past the oppressive sanctions imposed by the US government [15]. However, in 2019 the Iranian pharmaceutical market was about 2.50 billion USD [16].

Most pharmaceutical expenditures are imposed on patients and their families in the inpatient and outpatient sectors in Iran. According to the insurance law, in inpatient services, patients should pay only 10% of total pharmaceutical costs, and the remaining 90% must be paid by insurance. However, in reality, a much more percentage of the pharmaceutical costs is paid by patients. Because there are increasing pharmaceutical products out of the health insurance positive list, the insurance does not reimburse the cost of inpatient prescriptions dispensed in outpatient pharmacies. In addition, considering the strategy of insurance funds in covering the lowest priced available generic products, the patients would be persuaded to pay for the difference of covered products and uncovered one [13].

With these interpretations, good governance is, therefore, a sine qua non for ensuring better access to medicines [17], and it is currently a priority for the Iranian pharmaceutical system. Greater transparency in the pharmaceutical system will help to improve drug access [9]. Transparency in pharmaceutical policy decision-making is not an end in itself, but it is a tool to facilitate and encourage ethical practice and minimize corruption more generally [9, 10, 18, 19]. Governments need to know what areas of the system are less than optimal transparency and vulnerable to corruption. There is a need to monitor how pharmacies, hospitals, and health care providers are reimbursed for pharmaceutical products [17].

Transparency is ‘the degree to which access to government information is available [20–22]. ‘Understanding how decisions are made requires information about the procedures followed and the criteria used by policymakers to reach decisions’ [22–25]. ‘Understanding why decisions are made necessitates disclosure of the information drawn on by policymakers and revealing the arguments adduced in favor and against particular decisions’ [22, 26].

Several studies in developed and developing countries were conducted to assess the level of transparency and potential vulnerability to corruption in the pharmaceutical system. For instance,

Garuba, Kohler, and Hoysman [27] assessed the understanding of transparency and potential vulnerability to drug corruption in the four main areas of Nigeria’s pharmaceutical sector and showed that the country is prone to drug corruption. A study in 2009 evaluated different areas of Jordan’s pharmaceutical system, including registration, upgrade, inspection, selection, preparation, and distribution [28]. Another study investigated transparency to promote good governance in the public pharmaceutical sector in Lebanon in 2009 [28].

In the Syrian Arab Republic, the level of transparency and vulnerability to corruption in eight essential functions of the public pharmaceutical system was assessed [28]. In addition, Rosenberg-Yunger and Bayoumi [18] investigated transparency across 11 Canadian public drug advisory committees and claimed that the most important ways to improve transparency include creating formal appeal mechanisms, improving communication, and establishing consistent rules about the use of, and public access to, proprietary evidence. Badawi et al. [9] evaluated the level of transparency in Kuwait’s pharmaceutical sector and revealed that few functions of Kuwait’s pharmaceutical sector remain relatively vulnerable to corruption. Paschke et al. [8]provided a conceptual understanding of how transparency can facilitate accountability for better access to medicines.

Herandi et al. [29] evaluated the pharmaceutical regulatory sector of Iran from the aspect of transparency, quantitatively, and their study focused on good governance. It is inherently different from the current study. The current study is a qualitative study that evaluated the pharmaceutical sector of Iran profoundly and explored the challenges, the moral behaviors, and the solutions.

This volume of studies on this topic shows the importance of the subject. Because transparency in the pharmaceutical system firstly provides people with information about the benefits and harms of pharmaceutical products. Second, it leads to public trust. Finally, medicines transparency creates opportunities to improve decision-making and policy-making [30]. Unfortunately, despite the need for more, this issue is less addressed in LMICs.

So, in this qualitative study, the aim has been to assess the perceptions of pharmaceutical policymakers and other stakeholders about transparency in the pharmaceutical system of Iran as well as examines the transparency of Iran’s pharmaceutical sector in its eight main functions include: registration of medicines, licensing of the pharmaceutical business, an inspection of establishments and market control, medicine promotion, clinical trials control, selection of essential medicines, procurement, and distribution of medicines.

## Methods

### Study design

A qualitative study using a content analysis approach was employed to explore stakeholders’ views in the pharmaceutical sector about their perceptions of problems, immoral behaviors, and solutions related to transparency in the public pharmaceutical sector of Iran. This study was conducted using a semi-structured interviews method with a maximum variation of key informants in the public pharmaceutical sector. The study was carried out from 4 January 2017 to 5 March 2017.

### Sampling and participants

The study population included all key informants of public pharmaceutical policy in Iran. A maximum variation purposive sampling method with a snowball strategy was used to select and ensure a good representation of key informants at the national level. A total of 28 semi-structured interviews were carried out using a topic guide that incorporated the study’s objectives, including identifying the problems, the immoral behaviors, and the solutions related to transparency in each function of the public pharmaceutical policy sector in Iran. The topic guide consisted of a series of open-ended questions about the transparency in the public pharmaceutical sector, adopted from the standard assessment tool of the WHO called “Transparency Assessment Tool in Public Pharmaceutical Sector” [31].

### Data collection

Tables 1 and 2 show the sample population’s distribution, including the key informants across government, private sector, and academic settings. They were selected based on their knowledge about the subject and their level of involvement in the public pharmaceutical sector. The private sector workplaces including private retail pharmacies, local pharmaceutical manufacturers, and importers. The public sector workplaces including drug regulatory agencies (Food and Drug Organization related to the Ministry of Health, Treatment and Medical Education, Food and Drug department in Medical Universities), health insurance organizations, and academic experts. Before implementing the interviews, we provided a list of key informant groups eligible for the interviews. We found their contact details through their workplaces. Key informants were excluded if they canceled the interview meeting more than two times. Each interview lasted 60–120 min. The interviews were tape-recorded if the participants agreed with the written informed consent and then transcribed. All the interviews were recorded except in two cases, we took a note.

**Table 1:** Number of interviewees in each main functions in public and private sectors

Scope / Function	Frequency (percentage)		
	Public sector	Private sector	Total
Registration of medicines	13 (68.4)	6 (31.6)	19 (100.0)
Licensing of pharmaceutical business	14 (70/0)	6 (30.0)	20 (100.0)
Inspection and market control	14 (66.7)	7 (33.3)	21 (100)
Medicine promotion	12 (70.6)	5 (29.4)	17 (100)
Clinical trials control	12 (70.6)	5 (29.4)	17 (100)
Selection of essential medicines	14 (73.7)	5 (26.3)	19 (100)
Procurement of medicines	13 (76.5)	4 (23.5)	17 (100)
Distribution of medicines	16 (76.2)	5 (23.8)	21 (100)
Total participants	20 (71.4)	8 (28.6)	28 (100)

**Table 2:** the interviewee information

code	Sector	position	date
1	Private/public	Private retail pharmacy/academic expert	4 January 2017
2	public	drug regulatory agency	7 January 2017
3	private	Private retail pharmacy	8 January 2017
4	Private/public	Private retail pharmacy/academic expert	21 January 2017
5	public	drug regulatory agency	26 January 2017
6	public	drug regulatory agency/academic expert	18 February 2017
7	public	drug regulatory agency (Food and Drug department in Medical Universitiy)	21 February 2017
8	Private/public	Private retail pharmacy/academic expert	25 February 2017
9	public	drug regulatory agency	28 February 2017
10	public	drug regulatory agency	2 March 2017
11	public	drug regulatory agency	5 March 2017
12	public /private	academic expert /local pharmaceutical manufacturers/importer	7 March 2017
13	public	drug regulatory agency	11 March 2017
14	public	academic expert	15 March 2017
15	public	drug regulatory agency	9 April 2017
16	public	drug regulatory agency	12 April 2017
17	public	drug regulatory agency	16 April 2017
18	public	health insurance organization	18 April 2017
19	public	drug regulatory agency	20 April 2017
20	public	health insurance organization	23 April 2017
21	public	drug regulatory agency	29 April 2017
22	Private/public	Private retail pharmacy/ academic expert	2 May 2017
23	public	drug regulatory agency	14 May 2017
24	public	drug regulatory agency	16 May 2017
25	public	drug regulatory agency	27 May 2017
26	public	academic expert	3 Jun 2017
27	private	local pharmaceutical manufacturers/importer	10 May 2017
28	Private	Private retail pharmacy	13 May 2017

This tool consists of eight areas that evaluate eight functions related to transparency of procedures and structure of the country’s pharmaceutical sector, including registration, licensing, inspection, promotion, clinical trials, selection of essential medicines, procurement, and distribution. The methodology used in this assessment is intended to collect qualitative information by using open-ended questions. The researchers formulated an interview guide by applying that instrument and provided a list of questions to be answered by key informants.

The methods were performed under the relevant guidelines and regulations.

### Data analysis

Conventional content analysis with a hybrid approach with inductive data-driven and deductive approaches was used to analyze the data. In our study, we found the categories from the data flow. In this approach, researchers immerse in the data to allow new insights tofind, also described as inductive category development [32, 33].

### Trustworthiness

Data were mainly collected using interviews with open-ended questions. We also probed participants’ comments by asking open-ended questions such as ““Can you tell me more about it?”“For data analysis, two members of the research team (AE and HS) repeatedly read the transcribed interviews to achieve immersion and obtain a sense of the whole of the interviews. Then, they independently read the text of interviews word by word to identify meaning units and codes by highlighting the exact words from the text to capture critical thoughts or concepts. Then, the researchers approached the texts by making notes of the initial analysis. This process continued, and the labels for codes found as they reflected more than one key thought. Codes were then placed into categories based on how different codes are linked. Next, frequent sessions were held, and the research team reviewed, checked, and discussed the coded segments to verify the found codes and categories.

For reflexivity, at first, the researchers tried to suspend themselves in all stages of the research according to the Epoche and bracketing principle, especially in the data collection and analysis. This strategy minimized the impact of researchers’ perceptions and experiences on the research process and results. Nevertheless, considering the importance of context in qualitative research, researchers attempted to interpret the data related to the research context, i.e., Iranian society.

The researchers are faculty members of universities of medical sciences in Iran. They are graduated in health policy and economics. Their interests are health policy analysis, health economics, health care administration, pharmacoeconomics, pharmaceutical administration, and qualitative research.

It is clear that researchers have chosen the subject because of their research interest. Hoever in the process of the research, in order to control any kind of bias, they put their interests and views in perspective. Therefore researchers bracketed themselves to mitigate the potentially deleterious effects of preconceptions that may taint the research process.

## Findings

In this qualitative study, eight functions of Iran’s pharmaceutical system were explored in terms of transparency. The results related to each function were categorized in three sections: general problems, ethical problems, and solutions. The results are summarized and categorized in Table 3.

**Table 3:** Key informant perceptions on the pharmaceutical sector transparency

Scope/Function	General Problems	Ethical Problems	Solutions
**Registration**	-The generality of the laws -Incomplete and outdated laws -Conflict of interest	-Illegal payments -bribery	-Needs assessment -Clarifying conflicts of interest -Precise and detailed rules -Elimination of bureaucracy
**Licensing**	-Pharmacy monopoly -Centralism -Lack of consistency of processes -Conflicts of interest	conflicts of interest	-Transparency of laws -transparency of processes -Informing adequately - Breaking the monopoly (new productions)
**Inspection**	- Conflicts of interest -Legal weakness to support the inspector -Non-implementation of GMP	- Corruption	-Standardization -Inspector rotation -Declaring Conflict of interest
**promotion**	- Unlawful advertising - Conflicts of interest	-leisure trips offer	-Strategic purchasing -Advertising based on scientific evidence -Controlling physicians’ prescribing behavior -Requiring pharmacists to deliver generic medicines -Tracking effects of physician prescribing -Publicizing committee decisions -Clarifying committee criteria -Clarifying The standards -Develop a framework for advertising
**selection**	-Similar and multiple medicines Apply -Apply personal beliefs -medicine pharmacopeia list defect -Conflict of interest -Lack of essential medicines list	-Hidden powers to manipulating the list -Hoarding	- Transparency -Creating a local pharmacopeia
**Clinical trial**	- Failure to register and publish clinical trials - Non-registration of conflicts of interest		-Financial support -Sufficient and skilled manpower - Enhancing the power of clinical trial committees -Decentralization -Declaring conflicts of interest
**Procurement**	-Lack of transparent purchases -Imbalance power between companies - Conflicts of interest	-Dumping -Hoarding -Gifts (bribes) - Collusion and disputes between people	-National oversight system -Automation and online applications -Medicine tracking -Oversight of production
**Distribution**	-Corruption and misuse of subsidized medicines -Lack of electronic ordering system -Not Transparent medicine distribution -Lack of intelligent system -Lack of public reporting and performance reporting	- Companies gifts -Smuggling	-Transparent National Drug Supply and Distribution Information System -Designing of an online information system -Reduction of bureaucracy -preparation and distribution of high-quality drugs

### The first function: registration of medicines

#### General problems

The interviewees mentioned the many problems that the country’s medicines registration system faces. These problems include lack of attention to Health Technology Assessment (HTA) and health economics, lack of strategic purchasing, political and commercial incentives, lack of integrated registration process, irrational decisions, the power of pharmaceutical companies, lack of skilled workforce, personal taste, lack of transparency, and conflicts of interest.

##### The generality of the laws

The interviewees believed that the country’s drug registration system laws are so general that it is possible to exercise taste. Therefore, authorities act differently in the same situation. It is important to note that their actions are not illegal. Rather, the generality of the law causes these problems. This causes instability in the implementation of processes and procedures.

*"… It is a matter of taste, personal values* affect *evaluations. Some drugs are not allowed to be registered. Some drugs are not allowed to enter the market." (An official of the Food and Drug Administration (FDA)”*

*"… another person comes and acts differently; But, what he does is not illegal. Because the law is so general." (From the officials of the Food and Drug Administration)*

*"… sometimes, the new boss changes everything and acts differently. Because the law is so general, what he does is not illegal" (An official of FDA)*

*"… there are many problems in terms of lack of skilled manpower, lack of proper technology, lack of transparency and bureaucracy, and personal tastes*." *(An official of FDA)*

##### Incomplete and out of date laws

One of the important problems that the experts stated is that the existing laws are out of date and need to be completed and reformed. Existing laws have not been able to address all the problems and aspects. Some important issues may have been missed.

"*…There are some fundamental weaknesses in the Food and Drug Administration. The first weakness is that we do not have a comprehensive law. Our law is old. 1956, which is almost Sixty years ago, this law was amended two or three times in some articles of the government after the revolution. The law is old and not comprehensive. That is, it does not fully cover all aspects of medicine." (An official of FDA)*

*"…since we implemented the generic system in Iran, we have seen an increase in registrations." (An official of FDA)*

#### Ethical problems

Common problems identified in the country’s drug registration system in interviews include illegal payments, gift-taking, bribery, conflict of interest, and rationalism to medicine registration and importation.

*"Although some can register their medicine and easily enter the country, many others will not be able to do so that is favoritism in the pharmaceutical sector "(an official of Food and Drug deputy of the University of Medical Sciences (UMS.*

*"… I think the serious thing is dealing with conflict of interest. Because it is not clear. A person may be in a position of interest…They sometimes do not declared their conflict of interest, honestly" (An academic expert).*

#### Solutions

Interviewees suggested solutions to the problems of the drug registration, including precise and detailed rules, declaring conflict of interest, elimination of bureaucracy and registration of medicines based on need assessment,.

##### Need assessment

It was suggested that needs assessment criteria be used to control the quantity of drug registration. According to the data of interviewees, implementation of HTA, registration of cost-effective drugs, strategic purchasing, revising the essential drug list, approval by professional associations, organizing and strengthening the generic drug program are helpful in this way.

*"I came up with criteria and registration procedures to assess the needs of the country. One of the problems that the pharmaceutical sector faces, is a large number of registered drugs more than the needs of the country. Different companies register it, costs goes up, and finally the advertising issue arises "(An academic expert).*

##### Clarify conflicts of interest

In all interviews, it was clearly stated that the conflicts of interest were not taken into account. The solution suggested by the interviewees to solve many of current the problems of the drug registration system is to clarify and declare the conflicts of interest by individuals. The suitable criteria for membership in the professional commissions are required.

*“If I were in a position and I really wanted to think to register, I would first make the conflict of interest issue very clear, which is extremely important." (An academic expert)*

##### Precise and detailed rules

The suggestion was that the rules be written in such detail that no one could bypass them. Experts believed that the rules should not be such that everyone could act differently. *“With precise and detailed rules, they cannot do everything they want. When I was in the position, I tried very hard for the rules and regulations to prevail and eliminate personal tastes.” (Former senior officials of FDA).*

##### Elimination of bureaucracy

Interviewees suggested eliminating unnecessary bureaucracy and administrative procedures as much as possible. They stressed that complex bureaucracies slow down the processes.

*"First, I removed the registration bureaucracy as much as possible"(An official of Food and Drug deputy of UMS).*

### The second function: licensing of pharmaceutical business

#### General problems

General problems reported by experts in the field of registration in Iran include centralism, lack of consistency of procedures, conflicts of interest and pharmacy monopoly.

##### Pharmacy monopoly

One of the problems is that new licenses to establish pharmacies are difficult to issue and the laws favor existing pharmacies and create a monopoly in the market. According to the information obtained in this study, the competition in the field of medicine is in favor of large and experienced companies. In fact, obtaining a license for new companies is difficult. These conditions indicate a monopoly in this market.

“*In other words, the pharmaceutical market turned into a pharmacy for pharmacists, so they pressure to get a pharmacy license. Some laws such as Inheritance argue that a person who dies and owns a pharmacy can inherit a pharmacy license from his or her own heirs. This in itself creates controversy. As a result, a kind of serious reforms are necessary in my opinion* "(An academic expert)

##### Centralism

One of the problems explains the centralized drug registration processes at the national level. The interviewees believed that this centralism would slow down the process of licensing. In fact, the provinces are not allowed to check the application for drug licenses, and this is entirely within the authority of the Food and Drug Administration of Iran, which is affiliated with the Ministry of Health.

"*…their most important problem is that they are centralized, that is, they are only at the ministry level and are not delegated to medical universities in provinces*" (An official of Food and Drug deputy of UMS).

##### Lack of consistency of procedure

Interviewees stated that there is no fixed process for issuing licenses and that frequent changes occur. The reason for these changes is related to apply the personal taste of managers. They believed that this situation would lead to uncertainty and a lack of transparency.

"*This matter of licenses changes in different periods with personal tastes. For example, although one boss limits the number of licensing, the other one does not. That is, there is no clear instruction for limiting the number of licensing* “(An official of Food and Drug deputy of UMS)

##### Conflicts of interest

Interviewees repeatedly emphasized that one of the main problems in licensing to pharmaceutical companies is related to conflicts of interest. Many FDA officials also own pharmaceutical companies. This is a very problematic issue.

"*… Many who are involved in these legal mechanisms may themselves have interests, have companies, i.e. enter or not enter*" (An academic expert).

The suspicious licenses, the multiple licenses to similar medicines, licensing of low-quality medicines, power, inconsistent regulations, nontransparent process and selection criteria for the members of commissions, conflicts of interest, monopoly, and bureaucracy.

"*Our problem is that our law in medicine is very general*" (an official of FDA).

#### Solutions

The interviewees suggested solutions to licensing problems to pharmaceutical companies including transparency of laws, transparency of processes, registration of local medicines over imports, and giving information are needed. Breaking the monopoly of the pharmaceutical market through the issuance of new licenses was identified as a solution. We also need more transparency and review of conflicts of interest.

“*...If someone wants to have the conditions to establish a company, he should know the necessary information about the scope of company's activities”* (An official of Food and Drug deputy of UMS).

### The third function: inspection and market control

#### General problems

The interviewees mentioned the general problems in supervision and inspection of pharmaceutical companies, including non-evaluation of medicine prescriptions pattern, non-inclusion of Good Manufacturing Practice (GMP) standards in the supervision checklist, conflicts of interest, inadequate supervision, overlapping of Food and Drug Administration activities with other national organization for supervision, legal weakness to discipline, lack of qualified inspectors, lack of guidelines and standards, lack of transparency, personal tastes and corruption.

“*I’m sad that so much corruption has been occurred in these few years because there is no rule. You see, a rule should have existed. There is no such code of ethics. Some realized that giving and receiving gifts is the way to improve their job positions, so they corrupted the system. You see, this corruption is not related to drug and food, this corruption has existed in all sectors*" (An official of FDA).

*“Firstly, the number of inspectors is few. It is not proportional to the volume of pharmaceutical services actually in terms of production, distribution, and pharmaceutical services and there is no standard checklist in the field of control and inspection* ". (The Vice-Chancellor of FDA)

##### Conflicts of interest

One of the main problems mentioned in the interviews and was repeatedly emphasized was that the Food and Drug Administration inspectors are the personnel of the same companies that they are going to inspect. In other words, the inspectors are financially affiliated with the inspected pharmaceutical company. This issue creates substantial conflicts of interest. Also, the criteria for selecting inspectors are not clear.

"*Another serious problem that is being discussed is that every technical producer in the country should have a technical supervisor. The inspectors are financially dependent on the person they are inspecting, and of course, I think the term they use is not an inspector, but it is related to the role of the supervisor. Because the financial system of the FDA cannot afford this payment to inspectors. This technical supervisor is practically the representative of the legislature. This creates severe conflicts of interest. It means that it is natural for a person to be worried about his job. The most important thing is conflicts of interest* “(An academic expert).

##### Legal weakness to support the inspector

According to the interviewees, one of the general problems in the field of supervision is the lack of legal protection for inspectors. Unfortunately, it has been repeatedly stated that there are still no rules for many issues. For example, there is No inspection of manufacturers after licensing. Such weak regulation severely damages the functioning of the pharmaceutical system. Even some of the corruptions mentioned above can be due to the lack of complete and comprehensive laws.

"*You see, to inspect well, first of all, the laws that actually support the inspection must be well documented. We are weak in this area as well. There are not complete laws. We see weakness in this area need to pay attention” (An official of FDA).*

"*…The issue of inspection and control of post-marketing is probably talking about the manufacturers. However, in any case, our post-marketing is very weak. After the drug that enters the market should check by the Reference laboratories or drug control laboratories, unfortunately, they do not work accurately. They do not report quality. For example, a certain drug does not work, but you do not have any official report from the Food and Drug Administration that this drug finally works or does not work " (An academic expert).*

##### Non-implementation of GMP

One of the main problems mentioned by the interviewees is the non-implementation of GMP in Iran’s pharmaceutical system. Therefore, the inspection task is not performed properly.

Because one of the main aspects of inspection of pharmaceutical companies is ignored. This issue also refers to the weakness of the laws. This means that the GNP is not on the legislative agenda.

"*As far as I know, we do not implement GMP seriously in the country. That means we have some serious problems in the field of market control for various reasons. One is that our manufacturers did not exceed GMP standards, so our serious challenge is to discuss GMP when you do not meet GMP standards...*." (An academic expert).

#### Ethical problems

##### Corruption

Ethical problems in the area of supervision and inspection identified in the interviews included Corruption, immoral behavior, and ill-treatment with inspectors in the companies. Unfortunately, our findings show that all kinds of corruption can be seen in the pharmaceutical system of Iran. Types of corruption such as giving bribes to inspectors, giving gifts openly, etc. Of course, according to experts, the existence of this type of corruption is not specific to the pharmaceutical system but can be seen in the administrative systems of other ministries. According to the interviewees, all kinds of corruption have become a tool for personal growth and development in organizations.

“I’m sad that so much corruption has been occurred in these few years because there is no rule. You see, there should be a rule. There is no such code of ethics. Some realized that giving and receiving gifts is the way to improve their job positions, so they corrupted the system. You see, this corruption is not related to drug and food, this corruption is related to all sectors”. (An official of FDA)

"*Some officials of pharmaceutical companies have inappropriate and immoral behavior of inspectors*" (An official of FDA).

#### Solutions

To reduce the damage and problems in the field of inspection of the pharmaceutical system of Iran, several solutions were suggested by experts. These strategies included changing techniques, peers inspections, multiple inspections, and precise criteria.

##### Standardize inspection tools

Interviewees noted that the current inspection tool is not a standard and appropriate tool and needs to be standardized. Standardization of inspection processes is required. It is necessary to define checklists and detailed tools. Then create an appropriate software to enter information, store and synchronize it. Different inspectors must use the same software. Finally, if the institution status is approved by all inspectors, a confirmation will be issued.

*"… we must have a transparent tool; a standard tool such as a questionnaire or checklist for the inspection process. An inspector does not always inspect all of our hospitals. Different people inspect with different perspectives and tastes. We need to standardize the questionnaires used in the inspection “.* (An official of Food and Drug deputy of UMS)

*"…the right selection of inspectors is very crucial. They should be trained. I tried very hard to create a checklist and create a checklist form or electronically. Sending the report to a central computer, now 3 people report independently, then the average is taken, a certificate is issued for this visit. It is a major weakness. It is suggested that personal tastes eliminate and visit a factory twice a year and compare with each other. If a company could not adapt to standards, close its production line. We could not do this before, because of slow processes and weak coordination"* (An official of FDA).

"*Rotation of inspectors is necessary. One set may be inspected 10 times by an inspector, while it should be inspected once and the next time must evaluate by another inspector* “. (From the Vice-Chancellor for Food and Drugs of the University).

##### Conflicts of interest transparency

Given the conflicts of interest, experts believed that a statement should be drafted to clarify the conflicts of interests.

“*In addition to inspecting a place several times, it may become a well-defined relationship with a particular company*". (An official of Food and Drug deputy of UMS)

### The fourth function: Medicine promotion

#### General problems

The interviewees described the general problems that the country faced in the field of drug promotion. They stated that there were no written rules and regulations regarding medicine advertising. Lack of scientific comparison of medicines, commercial advertisements, visitor advertisements, case-based decisions, multiple stewardship, insufficient regulations or supervision, non-scientific advertising, and conflicts of interest are the most important issues. All of the above are related to poor regulation.

##### Unlawful advertising

Interviewees noted that although medicine advertising is banned in the country, pharmaceutical companies present their medicine to doctors in offices. In some cases, they do this advertising in some magazines.

*“In advertising, the medicines are directly promoting to doctors. Inviting doctors to a conference and promoting medicines to them. An international conference that is held on the sunny beaches of a country and you can come with your family for two weeks” (A health insurance expert).*

"… *You see, in Iran, direct advertising of medicines is a violation; Although, there is a law that implemented and enforced, some pharmaceutical companies avoid this and do the trick. For example, they sell the health magazine to the public and try to advertise the medicine or they send advertising to the doctors via short message system (SMS) and the content of these advertisements is not monitored*" (An academic expert).

##### Conflicts of interest

There was a conflict of interest in advertising corporate drugs to physicians. Unfortunately, there are no specific rules and regulations to prevent this. There is a ban on drug advertising in the Iranian media, except for the specialized medical journal. However, advertising is done in person in doctors’ offices and medical institutions.

"… *I have to express my conflicts of interest. I have to say if I am a consultant for a company. According to WHO rules, committee members must express all their conflicts of interest. Do we? Do people know the members of these committees in the Ministry of Health? Frequently it’s a secret* " (an official of Food and Drug deputy of UMS).

#### Ethical problems

Ethical problems mentioned by the interviewees include incentives and motivational tools, material gifts to doctors from pharmaceutical companies, and purposive leisure trips offer.

Unfortunately, all kinds of stimulants are secretly offered to therapists, prescribers, staff, and medical institutions. There is no clear oversight mechanism and judicial follow-up for this area. One of the most unethical problems in drug advertising, which according to the interviewees, occurs frequently, is drug advertising occurs abroad under the pretext of attending scientific conferences.

*"... They are given material gifts, there are irrational connections, not just between companies, even in laboratories, paramedics, hospitals. For example, while a doctor contracts with a laboratory, for every prescription, 15% of the fee must be returned to him. I mean, if I ordered an MRI, you have to give me 15% of the money. We do not have a specific rule “. (*An official of FDA*)*

*"… Some conferences have no scientific value, but a meeting is held by a company in a holiday destination, for example in Paris with providing a five-star hotel and material gifts only for promotion" (*an official of FDA*).*

#### Solutions

The solutions provided by the interviewees included strategic purchasing, evidence-based advertising, evaluate physicians’ prescription behavior, deliver generic medicines, prescription tracking, publicizing committee decisions, developing the standards, criteria for membership, and stewardship, and finally developing a framework for promotion.

"*First of all, a framework must be formulated for advertisements and promotions. If someone has a claim on a drug, he/she should present scientific documents and pieces of evidence*" (An official of FDA).

### The fifth function: clinical trial

#### General problems

##### Failure to register and publish clinical trials

One of the problems that the interviewees expressed about the country’s clinical trials was related to non- registration and non-publication of clinical trials. There is no specific system for recording all clinical trial studies. The main problem is that the results of these case studies are not related to the final pharmaceutical products.

*" the results of clinical trials usually do not inform academics. If there is a clinical trial for a product with a document, it is much easier for us to present it in university academic and we can defend it. If those claim that the drug has a specific safety or efficacy problem, trials can be useful " (*An official of Food and Drug deputy of UMS*).*

"*… We have internal regulations, which means that there is a mechanism for registering clinical trials. Everyone has to register it, but we do not implement our mechanism well* "(An academic expert).

##### Non-registration of conflicts of interest

*Another common problem mentioned in interviewees' conversations in the field of drug clinical trials were the issue of non-registration of conflicts of interest.*

"*Again, there is a matter of non-declaring of conflicts of interest. I repeat, conflicts of interest, as a serious issue must be declared*" (An academic expert).

#### Solutions

The solutions stated by the interviewed experts to solve the problems of the country’s clinical trials include financial support, sufficient and skilled manpower, of the clinical trial committee, enhancing the power of clinical trial committees in Food and Drug Administration, decentralization, and conflicts of interest.

" *Two or three well-trained experts should be select for doing this, and they should receive Continuing Professional Development. The clinical trial team should be regional in the country. A clinical trial should be conducted in every area through academic connection* " (an official of Food and Drug deputy of UMS).

### The sixth function: selection of essential medicines

#### General problems

According to the Interviewees, general problems of the country’s drug selection system include lack of a list of essential medicines, lack of an up-to-date list, lack of guidelines for drug selection, and lack of cost-effectiveness analysis and Health Technology Assessment (HTA).

*" cost-effectiveness analysis and measures are very crucial and have not yet been implemented in our country"* (An official of FDA).

"*We do not have economic mechanisms; we do not implement HTA in practice*" (An academic expert).

##### Similar and multiple medicines in the list

Another problem mentioned by the interviewees for selecting drugs in the country was the inclusion of similar and multiple drugs in the list. Having similar drugs is not a problem in itself. But when the comparison and selection of these drugs are not based on scientific evidence. It is a big problem.

" *our list is not indication-based. For example, suppose that a special drug is registered for blood pressure, whenever the second drug with the same efficacy wants to register, it should be able to discard the first drug with strong evidence (such as more efficacy or less side effects) and replace it. We are just a one-way road to our country's drug list. Nothing is removed from the list, only the medicines added*" (an official of FDA).

##### Apply personal beliefs

Interviewees also pointed to the problem of applying personal beliefs by officials in the field of drug selection. Unfortunately, there is no list of essential drugs in Iran at all. There is only a list of drugs covered by health insurance organizations. The main problem is that there are no clear and precise criteria for the inclusion of drugs in the list of health insurance in Iran. Personal interests and tastes are influential in finalizing the list.

*“Sometimes, it is a personal taste. Although a minister motivates to enter a vaccine, the other did not allow it to be included in the list, because that was not cost-benefit and cost-effective*(An official of FDA).

"*…. The list of essential medicines is different from the list of medicines covered by the insurance companies. We do not have a list of essential drugs in Iran. We have a list of drugs covered by insurance ". (An academic expert)*

##### Medicine pharmacopeia list defect

In the previous topic, information about the country’s drug list was mentioned. It should be noted that this list is not exhaustive. In addition, the list is not updated promptly on time. These defects make the medication list unable to meet all needs.

*"The most important thing a policymaker in Iran should do, in my opinion, is to create a list of essential drugs, there is nothing more important than this list. We neglected it”*(An academic experts).

##### Conflicts of interest

Interviewees, as in other areas for drug selection, mentioned the problem of conflicts of interest in the drug list. They also stated that members are not asked to mention conflicts of interest and are not registered anywhere. Therefore, conflict of interest is effective in determining the list. This effect is certainly not to improve people’s health.

*"…if you have a particular specialty in the specific drug and you also have an essential position, specialized drugs may be covered a lot and other drugs may not be"(*Academic expert).

##### Lack of list of essential medicines

One of the interviewees’ problems as a serious challenge are that there is no such thing as a list of essential medicines in Iran. The lack of a list of essential drugs means that policymakers and decision-makers in the country’s pharmaceutical system do not believe in providing a prioritized list of drugs.

"*…. The list of essential drugs are different from the list of drugs covered by insurance. We do not have a list of essential drugs in Iran. We have a list of drugs covered by insurance "(*academic expert).

#### Ethical problems

The ethical problems in the field of selection in the country include; the influence of specific individuals or groups for inclusion a medicine in the list, as well as hidden powers to enter or remove medicines from the list.

*" I mean that there are pressure groups with authority and hidden powers can allow a product to enter the country's pharmacopeia very easily or not allow”(*an official of Food and Drug deputy of UMS).

#### Solutions

Interviewees generally suggested solutions, including preparing a list of essential medicines, building a committee representing all stakeholders, and preventing personal tastes.

##### Transparency

One of the solutions presented by the interviewees was to make transparent and make available to the public all the country’s drug selection process steps.

*"The most important thing a policymaker in Iran should do, in my opinion, is to create a list of essential drugs, there is nothing more important than this list. We neglected it”* (An academic experts).

"*If I were the head of the organization, I would make all my processes transparent, and I would put them on the organization's website” (An official of FDA).*

##### Creating an essential drugs list

Another solution presented by the interviewees was to create a local pharmacopeia and force doctors to follow its pharmacopeia. Different regions of the country do not have equal access to medicines. Some drugs are only available in provincial capitals. Other cities may be deprived. A defined medication list should be provided for all areas.

*"The most important thing a policymaker in Iran should do, in my opinion, is to create a list of essential drugs, there is nothing more important than this list. We neglected it”* (An academic experts).

"*The national pharmacopeia is the most important matter for paying attention. We must localize it at first. For different provinces, for different regions, based on their burden of diseases, a pharmacopeia is needed. Secondly, implementing the pharmacopeia is much more important than developing the pharmacopeia. That is a weakness for the country*” (An official of the Vice Chancellor for Food and Medicine).

### The seventh function: procurement

#### General problems

Problems mentioned by the interviewees regarding the field of procurement included limited public resources, lack of drug tracking systems, strategic purchasing, transparency in drug distribution considering rationalism, and a comprehensive database for decision making.

##### Lack of transparency of purchases

Interviewees noted a lack of transparency regarding the purchase of drugs. For example, sometimes the amount of actual medicine purchased is different from the approved amount. In other words, the amount of drug delivered may be different from the amount recorded.

"One of the most *problems in the field of purchasing and supplying medicine is lack of transparency. How much medicine is delivered to the country's pharmaceutical system? Who is provided the medicine? And how are the medicines distributed? These facts in our pharmaceutical system should be transparent*" (An official of Food and Drug deputy of UMS).

##### Power imbalance between companies

Some interviewees pointed out that there are old companies with more power than others and can determine the desired profit, which has caused problems.

*" Supply and distribution of medicine is influenced by the old companies that enter the market, they themselves have long been the head of buying and selling the country's medicine, a firm with market power tend to affect the quantity and price”.( An official of Food and Drug deputy of UMS)*

##### Conflicts of interest

One of the major problem that the interviewees highly emphasized were the existence of conflicts of interest.

*"We have serious problems in this debate. The Ministry of Health has very, very serious problems with distribution. Unfortunately, it is not solvable either because the decision-makers, those who play a key role in the pharmaceutical system. They make decisions and distribute them around the drug system" (officials of the FDA of the university).*

"*Conflicts of interest is a serious problem in this scope. Unfortunately, sometimes it is not solvable because the decision-makers play a key role in the procurement and distribution scope in the pharmaceutical system*". (An official of Food and Drug deputy of UMS).

#### Ethical problems

Ethical problems identified according to the interviewees included dumping, drug hoarding, collusion, lack of fair rationing, supplier disputes, and bribery.

*“For example, dumping (a kind of injuring prices) may happen to defeat a domestic producer. When they are knocked out, they may raise their prices too much. These are all immoral things …"* (Private Sector Representative).

*"Immoral behaviors (with a laugh) in the field of drug procurement, we have people who are distributing drugs in the country, there are those who take the medicines in a specific direction, have a special goal, and do not distribute it. They want to distribute the medicines if the price increase, they are hoarding" (An official of Food and Drug deputy of UMS).*

#### Solutions

General strategies suggested by experts in the field of drug procurement included competitive bidding procedures by hospitals, medicine tracking, online applications, preparation and distribution of high-quality medicines, and strengthening the national production capacity.

"*The most important point is the transparency of the production system and medicine procurement and supply in the country*" (An official of Food and Drug deputy of UMS).

*“We should try to use the internal capacities, we really have the capacity of producing biotechnology medicines in Iran, considering all these professionals including biochemistries, biotechnologists, pharmacists, and specialist doctors. I went to a biotechnology company in Iran, I saw a very complex industry, it worked very well, and we are second in the world in these biotechnology medicines. It is not an exaggeration. Supporting domestic production is a successful policy that occurred in Iran so that we are not monopolized by other countries with very high prices. Later, clarifying the laws for preventing immoral issues is suggested* (an official of FDA).

Another solution was related to economic evaluation. Interviewees suggested conducting an economic evaluation before purchasing the drugs.

*“I believe in economic evaluation and pharmacoeconomics for procurement the medicines, the insurance companies should strongly evaluate the medicines before adding. I believe that for registration and procurement of medicines, efficacy, cost-effectiveness, and side effects must be evaluated; a medicine should be selected if it has the above criteria rather than existing drugs. I believe in these, but I do not believe in the essential list presented by WHO for Iran; that is 300 items of medicine*” (An academic expert).

### The eighth function: distribution of medicines

#### General problems

The interviewees mentioned the problems that exist in the country’s drug distribution system, which include corruption, pharmaceutical waste and expired medicine, abuse of subsided medicines, accumulation of medicines more than the consumption at the national level, medicine shortages, drug clearance, hoarding, Non-standard vehicles for pharmaceutical transport, purchasing of medicines for a long time due to time-consuming orders.

##### Corruption in use of subsidized medicines

Respondents believed that some of the drugs they were subsidizing had caused corruption and abuse.

*“We have subsidized medicines that distribute at a lower price such as drugs for special patients. An example of these drugs is insulin. Assume the real price of insulin is almost 2$, the subsidy price is 0.05$. Sometimes it is reported in a province 40,000 doses of insulin are sold to a distributor. In one item! Does a pharmacy in that province need 40,000 doses of insulin? You know that every diabetic patient needs about ten of these vials, and 40,000 doses mean 4,000 diabetic patients. It's just one item! It may distribute elsewhere*" (An official of FDA).

##### Lack of electronic ordering system

Respondents repeatedly pointed out that due to the lack of an electronic ordering system, many problems have arisen in providing adequate medicines. According to them, it is impossible to do.

*“There is a problem with the order point. You see, our most of our companies are weak at early taking orders, for example, in Tehran, if it works very well, it takes order once every two weeks. These orders should be electronic. If the software is available, pharmacies can receive medicine regularly, but this has not occurred. So because visiting are slow, they have heavy stocks of medicine* " (An official of FDA).

##### Not transparent medicine distribution

Lack of transparency on how much to distribute drugs among pharmaceutical companies was one of the interviewees’ ambiguities.

"*Next, it is not clear, how many medicines and how is it distributed in the country? Is it distributed fairly”?* (An official of Food and Drug deputy of UMS)

##### Lack of intelligent system

The interviewees stated that currently, the drug distribution system in Iran is facing the problem that there is no intelligent system that can immediately have accurate information about the country’s drug stock and in this regard reported the drug shortages or drug surpluses.

"*Here, I think we do not have an intelligent system that reports the average of drug stoke in the country. Medicine shortage or pharmaceutical surplus must report regularly. The country's inventory should not be lower than a certain point at any moment*" (An official of FDA).

##### Lack of public reporting and performance reporting

Another problem mentioned by the interviewees are that no performance reporting mechanism is defined for distributing pharmaceutical companies.

"*We do not have public reporting from distributors, which is a serious issue, we do not have performance reporting*" (An academic expert).

*“Whenever you need the medicine, you should be able to order it, but this is not occurred until now, so the pharmacies have heavy stocks of medicine, they have excess inventory, sometimes pharmacies have an inventory of 5 months which have a significant cost. For example sometimes, although they sell 11875$ monthly, they have 59375$ medicines in stock.”* (An official of FDA).

#### Ethical problems

Interviewees mentioned the ethical problems they face in the country’s distribution system, such as rewarding distribution companies, the relationalism of distribution companies, smuggling of drugs and counterfeit drugs.

"*Obviously, in the distribution process of drugs, smuggling drugs and trafficking are all issues that unfortunately may arise for pharmaceutical services both in terms of distribution and manufacturers* " (An official of Food and Drug deputy of UMS).

#### Solutions

The interviewees’ strategies included supervision of production and importing the medicines, facilitating the process of drug clearance, Reducing bureaucracy, and national monitoring system.

"*The most important thing is correct information in the pharmaceutical system. A proper information system of pharmaceutical services must be created that can be monitored, which can report as clear and transparent as possible what product has entered and how it is distributed*" (An official of Food and Drug deputy of UMS).

## Discussion

This qualitative study aimed to evaluate the transparency in the eight areas of the pharmaceutical sector in Iran, including registration of medicines, licensing of pharmaceutical companies, inspection and market control, advertising and promotion, clinical trials, selection of essential medicines, procurement, and distribution of medicines. This study is one of the first studies in exploring transparency in the pharmaceutical sector of Iran. This study is an initial step to increase the transparency and accountability of the public pharmaceutical sector. This study found several main results. The details of each result are discussed below. The challenges to the “registration” domain in the present study are the generality of the rules and outdated laws. Guidelines to limit registration and written guidelines on declaring conflicts of interest are also presented in previous studies’ results [18, 19, 27, 28].

In the “licensing” area, one of the challenges is related to the written and transparent criteria for selecting members of the licensing committee and written document describing the composition and conditions for referring to the giving licensing committee. Garuba et al. [27], in Nigeria study, explored there is an up-to-date list of registered pharmaceutical products in the country with the minimum level of information about the products. Documented procedures and standard forms exist and are publicly available for registration applications. There is formulated documentation with well-defined legal operating guidelines for assessors on how to process applications. Although there is a standing committee composed of directors of various departments for drug registration, there was no available documentation to support the selection and functioning of members of the committee that assesses applications. Additionally, there was no documentation to support the requirement of any specific professional qualifications, technical skills, work experience, or even research experience as criteria for committee membership.

Badawi et al. [9] evaluated the level of transparency in Kuwait’s pharmaceutical sector using the established assessment tool of WHO. They revealed that few functions of Kuwait’s pharmaceutical sector remain relatively vulnerable to corruption. The perceived strengths were the availability of appropriate laws, the presence of clear standard operating procedures, and the use of an efficient registration/distribution system and weaknesses were lack of conflict of interest guidelines and written terms of reference, absence of pharmacoeconomic studies, and inconsistencies in law enforcement . According to the study results, the situation of compliance with conflict of interest guidelines in Kuwait has been much better than in Iran.

In the context of Iran, in market inspection, writing update guidelines and regulations might be beneficial to manage and declare conflicts of interest appropriately. In Lebanon, In the “Inspections” area, a pharmacy law issued in 1994 covering the inspection of all pharmaceutical firms through an active inspection unit works on detecting counterfeit medicines and checking imported medicines at customs, and inspecting pharmaceutical firms [34].

Pharmaceutical guidelines generally address stakeholders upstream in the supply chain, such as manufacturers and distributors and specialists, including qualified persons, inspectors, and pharmacists [35]. According to (with) the evidence, there are some tools for the inspection. The World Health Profession Alliance published the Be Aware tool for visual inspection [35, 36], and the International Pharmaceutical Federation developed a similar tool [35, 37]. A simplified visual inspection checklist to allow field screening by healthcare workers is also developed at the point of care [35].

Pharmaceutical counterfeiting is becoming a serious problem both in developed and developing countries [38]. Public availability of audits results of drug regulatory agencies may provide an incentive for all agencies to ensure that their processes are well-documented and fully compliant with the guidelines and standards [27]. Hiring qualified personnel is very crucial in improving transparency. Shorts staffing can lead to corruption [27]. To reinforce transparency and accountability of the inspection process, it is also recommended to regularly rotate inspectors [27].

In the “promotion” area, the challenges included weakness in providing a process for following up the complaints regarding the reported immoral acts in drug promotion and the lack of a committee responsible for monitoring drug promotion. Lack of scientific comparison of medicines, commercial advertisements, and insufficient regulations. The results of a similar study in Papua New Guinea showed similar results [28]. It is suggested that clear criteria for selecting the members of such committee for the promotion of drugs should be written and publicly available. In addition, there are no written guidelines on conflicts of interest in control of drug promotion activities. This issue has led to the increasing growth of advertisement and promotions of therapeutic products, supplements, and beauty products without a precise mechanism in various media.

In the “selection” area, the most crucial issue is the lack of essential drugs necessary to respond to immediate medical needs in the country, even in any economic and social crisis. In Iran, although we have a list of essential medicines at the first level of health care, i.e., the rural family physician program, this list does not meet the precise criteria for selecting a list of essential medicines at all levels of health care delivery. There is also no comprehensive list with clear standards at the national level as required. Other challenges include weakness of explicit written and publicly available guidelines for the process of inclusion or exclusion of drugs from the list and low compliance of the list of essential medicines following the procedures and techniques of the World Health Organization, lack of clear criteria for selecting committee members, lack of transparency in terms of the role and responsibilities of the committee, and lack of standard written processes or operating procedures for the national committee decision-making process. In Jordan, the government officially adopted a national essential medicines list, the Jordan Rational Drug List, which helps the government to purchase appropriate medicines for their population in 2006 [28].

In the “procurement” area, the challenges are related to the written guideline for procurement, lack of specific criteria for membership in the committee, lack of written policies on conflicts of interest, lack of regular audits, and lack of management information system to report product problems in the procurement phase. Differences in drug procurement mechanisms at the national and local levels cause differences in transparency among countries. There are written guidelines for procurement office staff about procurement methods to be used for different types of products in Jordan. A formal appeals process is available for applicants who have their bids rejected. Additionally, there are clear and specific criteria for tender committee membership [28].

The process that favors local drug manufacturers could result in a more responsive and accountable industry that ensures a higher quality of the locally-made product would also enhance the nation’s economy [27].

Public procurement holds the most significant risk for corruption amongst all government functions. The risk of corruption in public procurement is well known globally [22, 39]. Substantial financial rewards from large procurement contracts lead to corruption [22, 40]. Corruption is not limited to a particular type of structure in a health system. It is present in all systems, including public or private, well or poorly funded, and technically complex or straightforward [22, 41].

In the “distribution” area, accumulation of drugs, hoarding, Non-standard vehicles for transporting drugs, the matter of expired medicines, and the purchase of medicines for a long time due to time-consuming orders are fundamental challenges. Transparent guidelines and monitoring are key strategies to increase transparency in this field. A similar study suggested that using video cameras and alarm systems in the distribution process would significantly enhance security and accountability for products [27]. In Jordan, the government has a transparent and explicit procedure for the distribution process for pharmaceutical products. Imprints on containers and external packaging can identify the public medicines. There are inventory records and techniques in the warehouse at various levels of the distributing system that are subjected to internal and external auditing. Sanctions are imposed on individuals for theft or corrupt practices. Although there are some strengths in that system, their weaknesses are that there is no adequate security management to oversee storage and distribution. There is no program for monitoring and evaluating the performance of the medicine distribution system [28].

Several studies conducted in other countries were mentioned above. Problems common to the findings of this study were observed in some areas. But in terms of determining the list of essential drugs, there was no such alignment. Most countries have generally implemented the WHO recommendation. A study in 2009 was conducted in Jordan using the WHO checklist and interviews with 61 key individuals [28].

Another study investigated transparency to promote good governance in the public pharmaceutical sector in Lebanon in 2009 in six functions of the pharmaceutical sector, including registration, inspection, promotion, selection, preparation, and distribution of medicines [34].

A study in the Syrian Arab Republic, the level of transparency and vulnerability to corruption in eight essential functions of the public pharmaceutical system was assessed [19].

Rosenberg-Yunger and Bayoumi [18] investigated transparency across 11 Canadian public drug advisory committees. They claimed that the most important ways to improve transparency include creating formal appeal mechanisms, improving communication, establishing consistent rules about use, and public access to proprietary evidence.

Paschke et al. [8] provided a conceptual understanding of how transparency can facilitate accountability for better access to medicines. They identified three categories of information as prerequisites for accountability: (i) standards and commitments; (ii) decisions and results; and (iii) consequences and responsive actions.

Herandi et al. [29] evaluated the pharmaceutical regulatory sector of Iran from the aspect of transparency, quantitatively. They claimed that to improve good governance, transparency should be maximized in all sectors, and this is possible by implementing mechanized actions and online tools. Medicines registration and distribution of medicines” scores in the 10- point rating system means that they are minimally vulnerable to corruption. Besides, medicine promotion control and procurement of medical products got an acceptable score, which means that they are marginally susceptible to corruption.

In this study, we found conflicts of interest as a shared problem in all eight areas. Also, the results of another study confirmed this finding. The biggest challenge of the pharmaceutical sector in Iran is lacking clear guidelines for conflicts of interest for every function represented in other studies [27]. Developing and implementing conflicts of interest guidelines can limit corruption. For example, Conflicts of Interest Guidelines for Common Drug Review (Canadian Agency for Drugs and Technology in Health Contractor) Approved December 2009. In this form, all participants must disclose any conflicts of interest, in the form and manner prescribed by the Canadian Agency for Drugs and Technology in Health, at the earliest opportunity [18].

### Limitations

This study used a qualitative method to identify the challenges and solutions of the public pharmaceutical sector, which has one limitation. Although we assured confidentiality of the information, two interviewees were unwilling to be recorded their voices during the conversation over corruption. To overcome this limitation, we took notes, instead.

### Strengths

This study is one of the first studies conducted in the field of corruption challenges in the public pharmaceutical sector at the national level. Although various studies in this field have addressed different aspects, this study conducted using a standard tool of the World Health Organization extracted a good framework for staging the pharmaceutical sector and the challenges of corruption at each stage.

## Conclusion

The present study is the first study that understands the different stakeholders of the health system of transparency in the country’s pharmaceutical sector. Most similar studies were performed quantitatively. Quantitative studies, despite being accurate, are unable to identify the depth of the problems. For this reason, in this study, the views of all stakeholders were analyzed by a qualitative method.

The results show that despite effective drug policies in the Iranian health system and government intervention, transparency in the country’s pharmaceutical sector is vulnerable. Corruption, lack of compliance with conflicts of interest protocols, and immoral behaviors are prevalent in the country. Some areas, including promotion selection and preparation of medicines, require necessary attention to review the policies. Iran’s pharmaceutical system should be compliant with the requirements of conflicts of interest. Formulation and implementation of conflicts of interest guidelines, Implementing Incentives and penalties system for compliance with policies can improve transparency.

## Supplementary Information

**Additional file 1.**

## Data Availability

The datasets used and/or analysed during the current study are available from the corresponding author on reasonable request.
