# Supplementary material for: Transparency in public pharmaceutical sector: the key informants’ perceptions from a developing country
Source: BMC Health Serv Res. 2021 Dec 7;21:1316. doi: 10.1186/s12913-021-07319-x (PMC8653549; doi:10.1186/s12913-021-07319-x)
Supplement: Supplementary file 1 — Additional file 1. [file 12913_2021_7319_MOESM1_ESM.docx]

Interview Guide

1. What are the problems in the “registration” area in the pharmaceutical system in the country?
2. What are the immoral behaviors that may exist in the country's pharmaceutical system in the “registration” area?
3. If you were in the highest-level position, what would you do to improve the country's pharmaceutical system in the “registration” process?
4. Can you tell me more about them?"

These 4 questions are asked in the interviews for all eight areas.
